# Harvest, After 50 Years of Sowing

**DOI:** 10.1007/s13659-018-0182-x

**Published:** 2018-07-19

**Authors:** Pema-Tenzin Puno

**Affiliations:** State Key Laboratory of Phytochemistry and Plant Resources in West China, Kunming Institute of Botany, Chinese Academy of Sciences, Kunming, 650201 People’s Republic of China

Professor Han-Dong Sun was born in November 1939 in Baoshan city, Yunnan Province. Over the past five decades since his graduation from Yunnan University in 1962, he has been engaged in basic and applied research on the exploration of plant-derived medicines and natural perfumes, phytochemistry and drug development (Fig. 1). He was systematically engaged in the study on more than 260 species of resources and secondary metabolites in China, including those of the genus *Isodon*, the *Taxaceae* family, *Schisandracea*e family, *Umbelliferae* family, *Lauraceae* family, and lichens. His work has facilitated the studies on natural products isolated from the genus *Isodon* and the *Schisandracea*e family to an international leading level. The detailed studies are as follows:

**Fig. 1:**
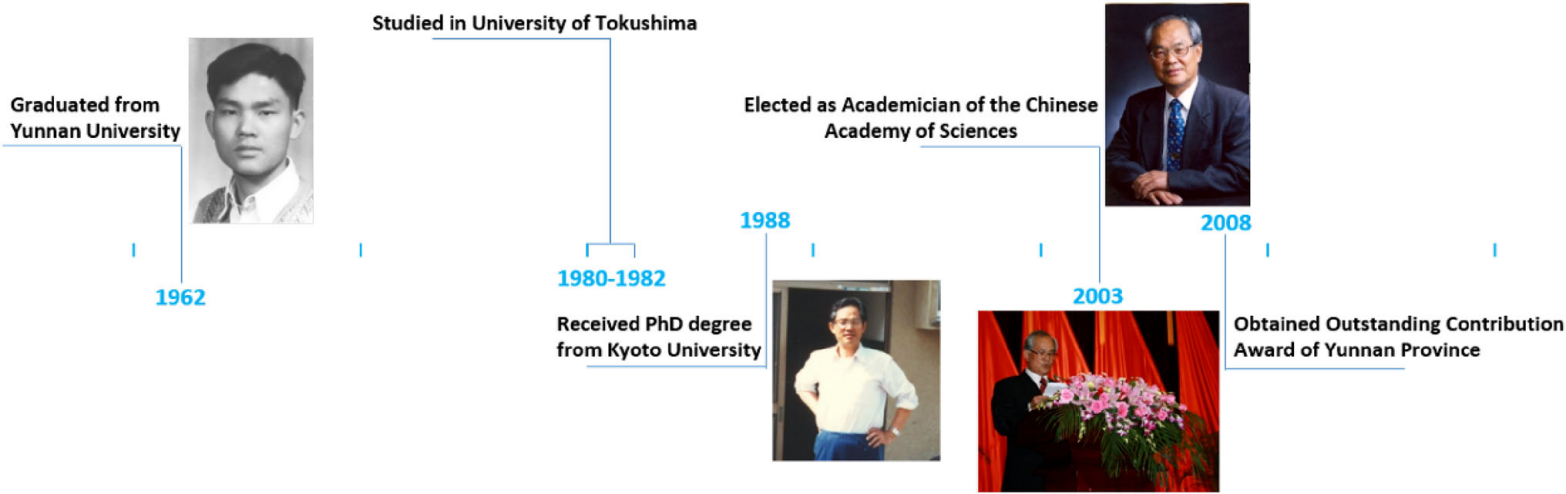
Milestones of Prof. Han-Dong Sun

## Research on Natural Perfumes in Yunnan

**Fig. 2:**
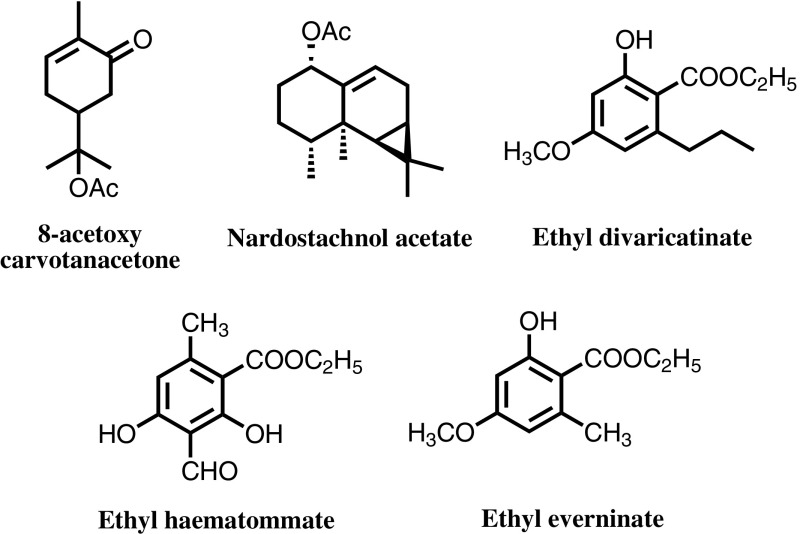
Representative chemical constitutes of natural perfumes

Han-Dong Sun was assigned to the essential oil group of a phytochemistry laboratory upon his arrival at Kunming Institute of Botany in October 1962. He purified and elucidated the chemical constituents contained in the essential oils of the plants in the genera *Cymbopogon*, *Cinnamomum*, *Litsea* under the guidance of Mr. Xian-Yuan Cai. His first research article in collaboration with Ren-Dao Lu, et al. titled “ The Volatile Composition of the Root of *Stelmatocryptin khasianum* (Kurz) H. Baill”, was published in “*Acta Pharmaceutica Sinica*” in 1963. From 1967 to 1972, he participated in a “523” Project, a civilian-military cooperation project for “Resisting America and Supporting Vietnam”, with his colleagues Jin-Kai Ding, Run-Bao Qin, Jin-Tian Yao, Cai Yu, Wen-Fen Chen, et al. Primarily run by the Institute of Military Medical Sciences, Kunming Military Region, the project was aimed at discovering mosquito-repelling constituents contained in plants. After over 5 years of military-civilian collaboration and exploration throughout many of the prefectures in Yunnan Province, they ultimately screened and discovered a new monoterpene called 8-acetoxy carvotanacetone (Fig. 2) from plant No. 1247, *Mentha haplocalyx*. The compound was successfully synthesized from *α*-pinene and possessed strong mosquito- and insect-repelling activities with no obvious side effects [1]. The project won the National Health Science Conference Award and the Second Prize of the Military Scientific Research Achievements in 1980. In the mid-1970s, in conjunction with Kunming Institute of Light Industry, they purified and synthesized nardostachnol acetate, a sesquiterpene with a persistent and intense aroma of sandalwood which is regarded an ideal raw material for rose- and fougere-type perfumes. The research provided a new sesquiterpene-based perfume for China, winning the First Prize of the Science and Technology Award of Kunming City and the Third Prize of the Scientific and Technological Progress Award of Yunnan Province in 1978 with the title “A New Perfume, Nardostachnol Acetate”. Moreover, their study of *Chinese angelica* resulted in the widespread application of *Chinese angelica* in medicine and perfume, creating a market for the angelica and its inferior product in Yunnan. The discovery was awarded the Third Prize of the Scientific and Technological Progress Award of Yunnan Province in 1985.

From 1982 to 1992, Sun’s group systematically probed into the aromatic and chemical constituents of 22 lichen species. They successfully developed two new lichen perfumery products, namely Chinese oakmoss No. 1 (with ethyl divaricatinate as the main aromatic component) and Chinese odour No. 2 (with ethyl haematommate and ethyl everninate as the main aromatic components) [2]. The aromas of these two products exhibited great similarities to those of French oakmoss products but are not identical. The perfumery project belonged to the National Ministry of Light Industry and Chinese Academy of Sciences during the period of the “Sixth Five-Year Plan”. Moreover, the project promoted the pioneering research and development of perfume in China. These products were praised both at home and abroad as original Chinese oakmoss products, successively winning the Second Prize of the Scientific and Technological Progress Award of Yunnan Province, “Sixth Five-Year Plan” Key Issue Recognition award of the Chinese Academy of Sciences, and Outstanding Product Award of Ministry of Light Industry in 1985 for its contribution to the perfume industry in China. In 1993, the project titled “Study on the Chemical Constituents and Application of Domestic Lichen Plants” was awarded the Third Prize of the Natural Science Award of the Chinese Academy of Sciences.

Since the late 1980s, Han-Dong Sun and his colleagues, Jin-Kai Ding, Xue-Jian Yu, Yu Wu, Yuan-Fen Yi, et al., under concerted efforts with colleagues Bi-Qian Chen, Xin-Xiang Ma, Yong Xu, et al., implemented a systemic study on essential oil contents, physicochemical constants, chemical constituents and primary aromatic components by means of GC/MS. Additionally, over the past decade, they reported the development and application of approximately 400 types of Chinese perfume plants, especially those found in tropical and subtropical climates, such as Dai Autonomous Prefecture of Xishuangbanna. The research provided a solid scientific basis for the comprehensive and in-depth understanding of perfume plant resources as well as their exploitation and utilization in Yunnan Province and throughout China. The results of the study were published in two monographs, which were “The Resources and Aromatic Components of Plants of Genus *Cinnamomum* in China” and “Perfume Plants and Their Utilization in Yunnan Province”, in 1997 and 2001 by Yunnan Science and Technology Press, respectively. These two monographs obtained high praise from perfume industry peers at home and abroad.

From the late 1970s to the 1990s, Han-Dong Sun wrote a number of reviews about the international progress of natural perfumes studies and the research progress and strategic discussion of perfume plants in both Yunnan Province and China. For example, “Progress in Analytical Research of Natural Aromatic Compounds” [3], “Perfume Plant Resources in Yunnan Province”, “Suggestions on Exploitation of Perfume Resources in Yunnan Province”, “Taking Full Advantages of Yunnan Natural Resources and Vigorously Developing Natural Perfume Production”, “Advances in Lichens Perfume” [4], “Natural Perfume Plant Resources” and “Chinese Perfume Plant Resources” [5, 6] have been published and well-received in both domestic and foreign publications. These publications boosted the research and development of natural perfumes in China and contributed greatly to the development of the natural perfume industry in China. It is noteworthy that “Study on Resource Exploration and Strategic Development in Southwest China” (containing the subproject “Natural Perfume Plant Resources” that was completed by Han-Dong Sun), sponsored by the Chinese Academy of Sciences and Natural Resources Comprehensive Investigation Commission of the State Planning Commission, won the Science and Technology Progress Award of the Chinese Academy of Sciences in 1993 and the Second Prize of the National Scientific and Technological Progress Award in 1995.

## Research on Medicinal Plants Such as *Taxaceae*, *Umbelliferae* and *Erigeron breviscapus*

**Fig. 3:**
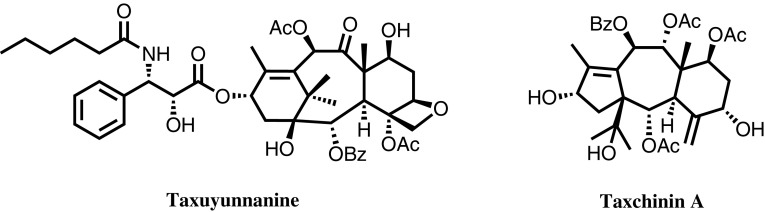
Representative chemical constitutes of *Taxaceae* species

Since the US national Food and Drug Administration (FDA) approved Taxol as a drug for the treatment of ovarian cancer in 1992, it has been a subject of great interest for natural product chemists and pharmacists worldwide. Currently, *Taxus brevifolia* is not widely available in China, but an abundance of *Taxaceae*, such as *Taxus yunnanensis* and *Taxus chinensis,* could be found in Yunnan Province. Sun’s group emphatically studied the aforementioned two plants obtained from different habitats jointly with Japanese researchers. Dr. Hong-Jie Zhang discovered a Taxol analog named taxuyunnanine (Fig. 3) in the root of *Taxus yunnanensis* [7]. The analog exhibited cytotoxicity against KB cells with the same order of magnitude as that of Taxol. Dr. Bo Li derived the taxchinins, a series of novel diterpenoids, from the leaves of *Taxus chinensis* [8]. These systematic and in-depth investigations on *Taxus yunnanensis* and *Taxus chinensis* provided a solid scientific basis for the rational development, utilization and protection of *Taxaceae* species in Yunnan Province.

**Fig. 4:**
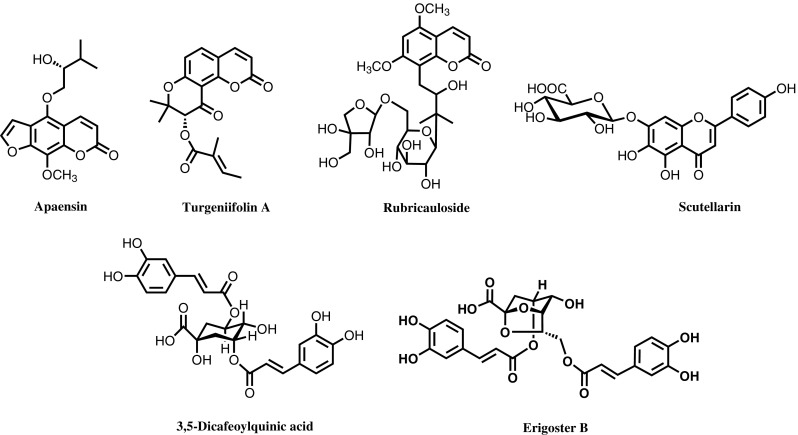
Representative chemical constitutes of *Umbelliferae* species and *Erigeron breviscapus*

Starting from 1974, Sun and his colleagues centered on the chemical constituents of *Angelica apaensis*, *Heracleum rapula*, and *Heracleum scabridum*, which are three plants of the *Umbelliferae* family. The results were released in “*Acta Botanica Yunnanica*”, “*Chinese Traditional and Herbal Drugs*”, and “*Bulletin of Botany*”, respectively from 1975 to 1978 [9]. Later, Sun’s group systematically explored the chemical constituents of more than 30 *Umbelliferae* species in southwest China. More than 300 compounds were purified and elucidated, among which more than 20 were reported for the first time, including apaensin, turgeniifolin A, and rubricauloside (Fig. 4), resulting in the publication of over 40 papers. These results not only increased the number of known chemical constituents and natural coumarins found in the *Umbelliferae* family but also clarified the association between their pharmacology and their chemical constituents. The research provided a scientific basis for the rational development and utilization of the *Umbelliferae* species. Of note, the project titled “Studies on Chemical Constituents of 13 *Umbelliferae* Species” won the Third Prize of the Natural Science Award of Yunnan Province in 1992.

Since the early 1990s, Sun and his group have spent more than 10 years studying on the bioactive constituents of *Erigeron breviscapus*, which is a well-known folk medicine for the treatment of cardiovascular and cerebrovascular diseases in Yunnan Province. In combination with biological screening, the previously reported compound, scutellarin, as well a variety of novel caffeoylquinic acid esters containing significant bioactivities were found, such as 3,5-dicaffeoylquinic acid and erigoster B (Fig. 4) [10]. Sun’s group also developed a Dengzhanxixin phenol drug (for injection), designated State Category II New Drug, which received clinical approval from the China Food and Drug Administration (CFDA) in December 2003, and completed its Phase III clinical studies in June 2007. The drug has been undergoing the new-drug certification process. Dengzhanxixin phenol drug (for injection) is a new preparation with clear effective components, controllable quality and a definite curative effect. Its unique effects for the treatment of cardiovascular and cerebrovascular diseases make this new drug a potential pillar of the biomedical industry of Yunnan Province, and one that possesses independent intellectual property rights in China.

## Research on Diterpenoids from *Isodon* Species

The genus *Isodon* (Labiatae, Ocimoideae) comprises approximately 150 species worldwide, with over 90 species and 21 variaties distributed in China, primarily in the southwest region. The greatest variety is found in Yunnan Province, with 46 species and 16 varieties. The identification of enmein by Japanese researchers in 1966 as an anti-bacterial and anti-inflammatory agent from *I. japonica* revealed *ent*-kaurane diterpenoids (or *ent*-kauranoids) to be the characteristic bioactive constituents of the *Isodon* species, a hot research topic in the field of natural product chemistry. In the 1970s, *I. rubescens* was utilized as tea by residents in Lin County, Jinan City, Henan Province, and was found to be effective for the treatment of esophageal and cardia cancer. Subsequently, a collaborative group was established in 1973 to investigate the anticancer components of *I. rubescens*, and Han-Dong Sun, et al. were invited to participate in the cooperation team in 1975. In 1976, rubescensin A (oridonin, or Ori) and ponicidin were identified by Han-Dong Sun, et al., and were proven to be the chemical constituent responsible for the bioactivity against esophageal and cardia cancer. The finding was reported simultaneously and independently by Japanese researchers. For over four decades, Han-Dong Sun, et al. have been engaged in the long-term, systematic and in-depth research on diterpenoids isolated from *Isodon* species found in abundance in our country, resulting in a series of major research findings. “Research on Diterpenoids and Their Anticancer Bioactivities from *Isodon* Species” is one of the breakthroughs made in the field of natural product chemistry and natural anticancer lead compounds. The systematic and innovative research provides a solid theoretical foundation and scientific basis for the rational utilization of the plant resources of the genus *Isodon* in China. The major research findings are as follows:

1. To date, 79 *Isodon* species have been phytochemically investigated by groups around the world, leading to the identification of 1048 diterpenoids, including 42 types of *ent*-kaurane diterpenoids and 28 novel skeleton types. Sun et al. carried out a study on the chemical constituents of 67 *Isodon* species and identified 732 diterpenoids (accounting for over 70% of the total amount) therein, including 30 types of *ent*-kaurane diterpenoids and 24 novel skeleton types. From these efforts, a series of lead compounds with potential for further development were identified. The project is remarkably systematic and innovative, which has made the research group a world center for research on the chemical constituents of the *Isodon* species and their bioactivities [11–20].
2. Research on the chemical constituents of the *Isodon* species has led to the discovery of a large number of new compounds. For instance, a rare and highly oxygenated *ent*-kaurane C-glycoside, neoadenoloside A, was found from *I. adenolomus*; two spirolactone *ent*-kauranes with novel scaffolds, neolaxiflorins A and B were isolated from *I. eriocalyx* var. *laxiflora,* and a new diterpenoid-based macrolide possessing a unique 10-membered lactone ring was isolated from *I. ternifolius*. The above findings have provided more diverse frameworks and increased the chemical space for subsequent research on novel chemotherapeutics (Fig. 5).

**Fig. 5:**
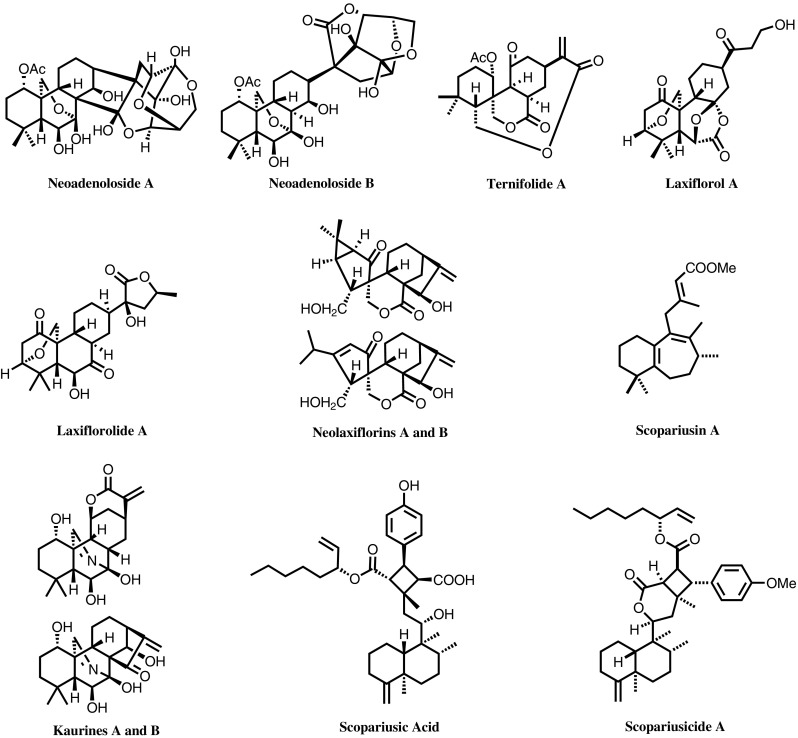
Representative novel diterpenoids from *Isodon* species
3. Pharmacological studies were performed on eriocalyxin B (EriB) and oridonin isolated from *I. eriocalyx*, demonstrating that eriocalyxin B and oridonin suppress tumor growth primarily through inhibition of the NF-*к*B signaling pathway, with the direct target identified as the p50 protein; in vivo studies indicated that EriB demonstrated remarkable growth inhibition of the breast cancer cell line MDA-MB-231. Pharicin A, isolated from *I. pharicus,* can induce mitotic arrest in paclitaxel-resistant cell lines (Jurkat and Raji), implying a different mechanism of action and offering a new approach for overcoming paclitaxel-resistant malignant tumors. Pharicin B, also isolated from *I. pharicus,* has been shown to stabilize the retinoic acid receptor-a (RAR-a) and synergistically enhances differentiation in several AML cell lines, particularly in some primary acute promyelocytic leukemia (APL) cell lines, when used in combination with ATRA. Furthermore, pharicin B can overcome the resistance in some ATRA-resistant cells. Adenanthin, isolated from *I. adenanthus,* can induce differentiation of APL cells by directly targeting peroxiredoxins I and II, revealing a new leukemia cell differentiation mechanism. The finding was selected as one of the “Top Ten Progresses in Chinese Science” in 2012 (Fig. 6).

**Fig. 6:**
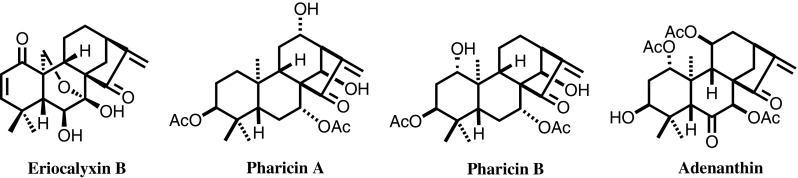
Representative lead compounds from *Isodon* species

Sun’s group works closely with pharmacologists at home and abroad to investigate the anticancer properties of diterpenoids isolated from *Isodon* species, achieving dramatical breakthroughs. With the knowledge that oncotherapy is gradually moving towards target-based and individualized therapy, *ent*-kaurane diterpenoids have broad prospects in their application as anti-cancer drug candidates. Based on the previous research work, “Donglingcao lozenges” and “Maoexiangchacai tablets” have been successfully developed as antibacterial and anti-inflammatory drugs that are particularly effective for upper respiratory tract infections. After being brought to market, they have proved economically rewarding and have created social benefits for years.

One hundred and forty papers have been published concerning this project, 124 of which were published in SCI-listed journals. Seven patent applications have been submitted, and 5 of them have been granted. Two monographs, “*Diterpenoids from Isodon species*” and “*Chemistry of Diterpenoid*s”, have been completed.

The project has been awarded the “Second Prize of Natural Science of Chinese Academy of Sciences” in 1992 and the “First Prize of Natural Science of Yunnan Province” in 2002 and 2013 respectively.

## Pioneering Research on Schinortriterpenoids

Schisandraceae is a family of important medicinal plant with two known genera, *Schisandra* and *Kadsura*. Our country possesses the most abundant sources of Schisandraceae species, and 21 of them can be used for medicinal purposes. For example, the dried mature fruits of *S. chinensis* have long been used in traditional Chinese medicine to enhance mental and physical working capacities and have been included in various editions of the Chinese pharmacopoeia. More than 50 years of prior studies on Schisandraceae species have revealed that their major secondary metabolites are dibenzocyclooctadiene lignans and lanostane- and cycloartane-type triterpenoids. Additionally, bifendate and bicyclol, two new anti-hepatitis drugs developed by Chinese scholars with independent intellectual property rights, have provided great economic and social benefits. However, owing to the previously insufficient research conditions, further investigations should be made about the chemical constituents of such group of important medicinal plants, the chemical basis underlying their medicinal uses, and the rational and sustainable exploitation and utilization of these abundant plant resources. Sun and colleagues have implemented systematic and in-depth research on the triterpenoids from 24 Schisandraceae species ever since early 1990s.

1. *Pioneering research on schinortriterpenoids (SNTs)* The most significant achievement was the discovery of highly oxygenated schinortriterpenoids of the *Schisandra* species, characterized by polycyclic-fused scaffolds containing numerous contiguous stereogenic centers (Fig. 7). These compounds have provided new clue for future development of pharmaceutical agents derived from the well-known traditional drug *Schisandra chinensis* and added important new dimensions to our understanding of triterpenoid chemistry.

**Fig. 7:**
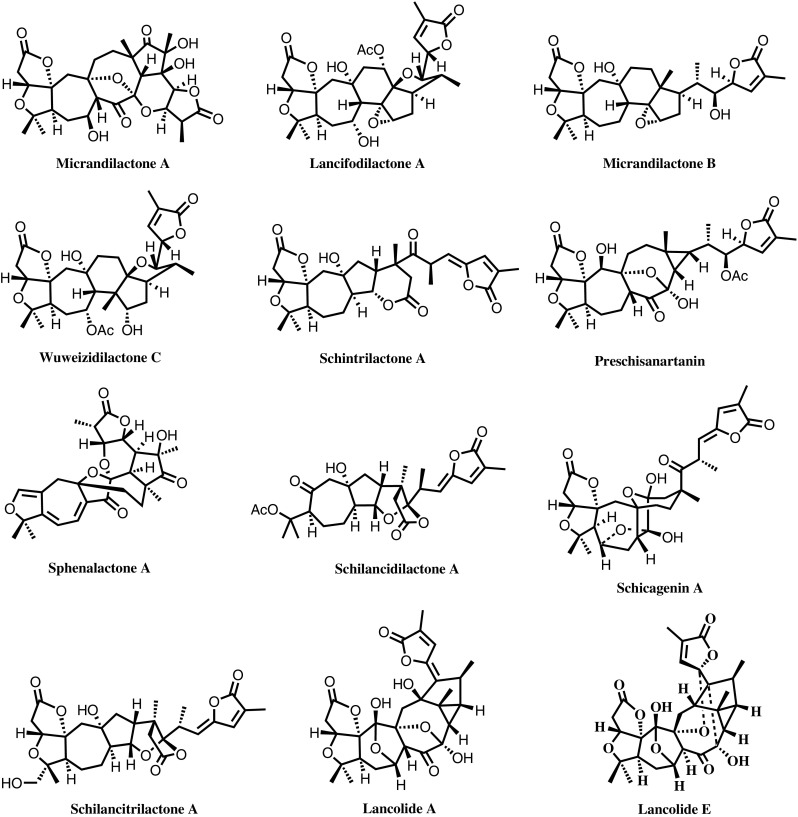
Representative novel SNTs from *Schisandraceae* species
2. *The discovery of triterpenoids with novel skeletons from the Kadsura species* two series of highly oxygenated triterpenoids possessing unusual ring systems and potent antitumor activities have been isolated from the genus *Kadsura*. The finding is a major breakthrough, which provides opportunities to identify new lead compounds and further investigate the genus.
3. *The total synthesis of schinortriterpenoids* the novel and complex structures of the schinortriterpenoids has aroused great interest among organic chemists at home and abroad. So far, 13 SNTs, including schindilactone A and lancifodilactone G, have been synthesized by 12 research groups in five countries, leading to the publication of over 30 papers in highly influential journals, including *J. Am. Chem. Soc.*, *Angew. Chem. Int. Ed.*, and *Nat. Commun.*

One hundred and fifteen papers concerning this project have been published in SCI-listed journals, including *Angew. Chem. Int. Ed.*, *Org. Lett.*, and *Chem. Commun*. Among them, 48 papers are ranked top 15% in our field. A number of these papers were cited by articles published in *Chem. Rev.*, *J. Am. Chem. Soc.*, and *Angew. Chem. Int. Ed.*

The discovery and synthesis of structurally novel and diverse SNTs has attracted the attention of natural product chemists, synthetic organic chemists and pharmacologists at home and abroad, and relevant studies have been cited in numerous reviews, textbooks and monographs. The work is an exemplary illustration of the research on the chemical constituents from one phytogroup. It has been recognized as one of the best achievements in natural product research by Chinese scholars who have become global leaders in the chemical studies of the Schisandraceae species.

**Scheme 1:**
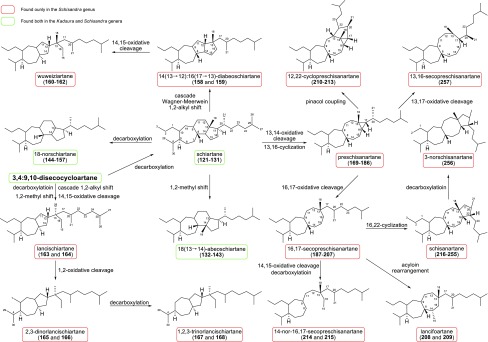
Proposed major biogenetic pathway, classifications, and distributions of schinortriterpenoids

In 2015, our group published a review on the latest research on the Schisandraceae triterpenoids in *Natural Product Reports* (NPR), in which the structural classifications, bioactivities, synthetic organic efforts, and the possible biogenetic pathways (Scheme 1) [21–45] were comprehensively depicted.

The systematic research on Schisandraceae triterpenoids is an original and symbolic research achievement made by Chinese natural product chemists that is recognized around the world. The research promotes the global academic status of Chinese natural product chemistry research and leads the development of phytochemistry and synthetic organic chemistry in specific fields. Therefore, “Chemical Studies of Schisandraceae Species” was awarded the “Special Award of Natural Science of Yunnan Province” in 2014.

As of 2017, the group has published 760 papers in peer-reviewed journals, including over 530 that were published in SCI-listed journals. Four monographs have been published, and 24 scientific research achievements received prizes from the Chinese Academy of Sciences, as well as from the state, provincial and ministerial level authorities. More than 70 doctoral and postgraduate students have been trained.

Since the implementation of Reform and Opening Up four decades ago, people have witnessed the rapid advancement of the Chinese economy. With strong support from the state and multilevel governments, the platform for natural product chemistry research in China has been substantially improved. Thanks to the efforts made by researchers, Chinese natural product chemistry research has transformed from the status of “following” as seen 40 years ago to a “neck and neck” position, even leading the way in particular fields. The abundance of plants and other natural product resources, in addition to the 2000-year history of the application of traditional Chinese medicine, is an inexhaustible gold mine for in-depth exploration. With continued clarification of our research directions and objectives, collaboration with other rapidly developing disciplines, consideration of national development needs and persistent research, I believe that we can make greater contributions and bring Chinese natural product chemistry research to a new golden era dominated by more fields. Based on the previous prospective results, I hope that our research group will broaden the horizons and keep pace with the development of modern natural product chemistry to supplement our achievements and finally contribute to the realization of the “new age” in our country.
